# Electromagnetic modeling of waveguide amplifier based on Nd^3+ ^Si-rich SiO_2 _layers by means of the ADE-FDTD method

**DOI:** 10.1186/1556-276X-6-278

**Published:** 2011-04-04

**Authors:** Christian Dufour, Julien Cardin, Olivier Debieu, Alexandre Fafin, Fabrice Gourbilleau

**Affiliations:** 1 CIMAP, CEA/CNRS/ENSICAEN/UCBN, 6 Boulevard Maréchal Juin, 14050 Caen Cedex 4, France

**Keywords:** Silicon, nanograin, Silica, Neodymium, ADE-FDTD, Waveguide, amplification

## Abstract

By means of ADE-FDTD method, this paper investigates the electromagnetic modelling of a rib-loaded waveguide composed of a Nd^3+ ^doped Silicon Rich Silicon Oxide active layer sandwiched between a SiO_2 _bottom cladding and a SiO_2 _rib. The Auxilliary Differential Equations are the rate equations which govern the levels populations. The Finite Difference Time Domain (FDTD) scheme is used to solve the space and time dependent Maxwell equations which describe the electromagnetic field in a copropagating scheme of both pumping (λ*_pump _*= 488 nm) and signal (λ*_signal _*= 1064 nm) waves. Such systems are characterized by extremely different specific times such as the period of electromagnetic field ~ 10^-15 ^s and the lifetimes of the electronic levels between ~ 10^-10^s and ~ 10^-4 ^s. The time scaling method is used in addition to specific initial conditions in order to decrease the computational time. We show maps of the Poynting vector along the propagation direction as a function of the silicon nanograin (Si-ng) concentrations. A threshold value of 10^24 ^Si-ng m^-3 ^is extracted below which the pump wave can propagate so that a signal amplication is possible.

## Introduction

The feasibility of optical amplifying waveguide has been for almost two decades the purpose of numerous experimental works [1]. The devices under study were based on an active layer constituted of a silica film co-doped with silicon nanograins (Si-ng) and rare earth ions RE (Er^3+ ^in particular) deposited on a substrate and covered by a cladding layer of pure silica. The differences in the optical indices of the three layers ensure the optical guiding. The amplification of a signal is based on an efficient population inversion of the rare earth levels whose energy difference correspond to the signal wavelength. Due to the very low RE signal absorption cross section, a solution has been found using silicon nanoparticles. The physical background lies on two major phenomena: on the one hand, the ability of Si-ng's to absorb efficiently a pumping light and, on the other hand, the effective energy transfer between Si-ng's and RE ions. In this way, a RE population inversion could have been achieved in order to fulfill the amplification function of the device. Despite all these promising features, a net gain is hardly achievable with the former Er^3+ ^ions due to their great probability of signal reabsorption from the ground state. This drawback is prevented with the use of Nd^3+ ^ions described by a five level scheme since the transition does not involve the ground state. The theoretical studies of the waveguide amplifiers have accounted for both rate population equations and Maxwell equations. In this paper we investigate the ADE-FDTD method applied to a rib-loaded waveguide whose active layer is composed of a silica film co doped with Nd^3+ ^ions and silicon nanograins. One of the main issues to be addressed in such systems consists in dealing with extremely different time scales: the populations lifetimes (1 ms) and the electromagnetic field period (10^-15 ^s). According to [2,3] we use a time scaling that allows to circumvent this issue. All the lifetimes have been shortened by a factor of 10^6^, and consequently the transfer coefficient K has also been divided by the same coefficient. In this paper, we investigate the accuracy of this scaling method through longitudinal and trans-verse maps of the Poynting vector for several Si-ng concentrations. The applicability of this method is linked to the space and time calculation steps since a reasonable computing time must not be exceeded.

## Computational details

**Figure 1:**
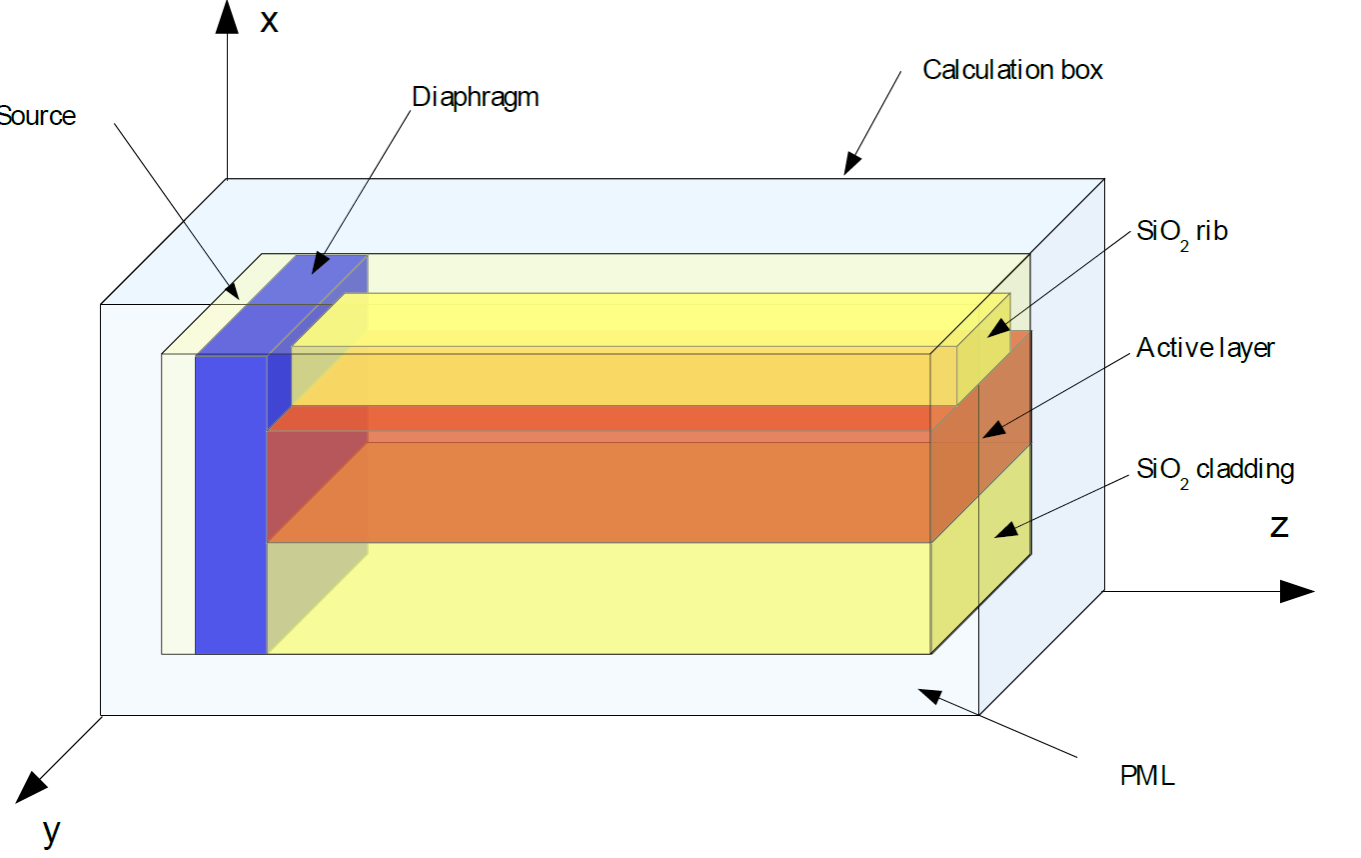
**Computing scheme**.

We treat the problem within a calculation box as described in Figure 1. Each axis (*x*,*y *and *z*) is divided into space steps (Δ*x*, Δ*y *and Δ*z *respectively).

Four zones appear and will be described hereafter: *i*) the rib-loaded waveguide composed of the active layer (optical index *n_act _*= *1*.*52*) stacked between the SiO_2 _cladding and rib (optical index ), *ii*) the plane containing the electromagnetic field source (*z_source _*= 6 Δ*z*), *iii*) the diaphragm (between 7Δ*z *and 10Δ*z*) which transforms the source into a realistic electromagnetic Gaussian field impinging on the waveguide and *iv*) the boundary zone (PML) (4Δ*z *in thickness) characterized by appropriate values of electrical (*ρ*) and magnetic (σ) conductivities in order to absorb the electromagnetic field so that the box borders do not influence the field in the zone of interest [4].

### Lorentz Model for the dielectric susceptibility

Considering a transition between levels *i *and *j *we use the Lorentz following relationship which makes the coupling between the polarization density **P***_i j_*, the level populations *Ni *and *N_j _*in *m*^-3 ^and the total electric field **E**:(1)

Δ*ω_ij _*is the FWHM of the *ij *transition deduced from photoluminescence measurements according to [5],  is the oscillator pulsation linked to the *ij *transition wavelengh *λ_ij_*,  and *γ_ij _*is the *ij *radiative transition rate in s^-1 ^[6,7]. The level populations difference in m^-3 ^is given by Δ*N_ij _*= *N_i _*- *N _j_*.

In the same way, we describe the polarisation density **P***_si _*linked to the silicon level populations *N_Si _*(ground level) and  (excited level),to the oscillator pulsation *ω_Si _*and finally to the transition FWHM Δ*ω_Si_*.

### Maxwell equations: FDTD numerical method

We start from the Maxwell equation which links the displacement vector **D **to the magnetic excitation **H**:

where the current density **J**_*e *_is related to **E **by **J**_*e *_=σ**E **where σ is the electrical conductivity. Accounting for the relationship between **D **and the total polarisation density, **D **= ε_0_**E + ∑ P_ij _**we may write:(2)(3)

All the calculations are performed with real variables. Hence, in order to account for absorption processes other than those due to the level transitions, we characterize (especially for the diaphragm and PML) a specific electric conductivity σ and magnetic conductivity *ρ*.

Both equations 2 and 3 are solved using the Yee algorithm[8]. The space steps are chosen so that: Δ*x *= Δ*y *= Δ*z *≪ *λ_min _*(the lowest values among all the wavelengths) Hereafter: Δ*x *= Δ*y *= Δ*z *= 45 nm. The time step Δ*t *must fulfill the condition: . Finally the fields inputs (pump and signal) are known as the 'source issue'. Since no perfect source is available, we choose an *xy *plane at *z_source _*= 6Δ*z *in which we define a polarized electric field. . This source impinges on the diaphragm so that a Gaussian beam enters the waveguide itself at *z *= *11*Δ*z*. The total waveguide length is 15Δ*z *= 0.665 *μ*m and the number of time steps is 25000, which amounts to a total simulated time of 0.3 10^-12 ^s.

### Rate equations

In this section, we detail the ADE part of the method which describes the time population dynamics of Si-ng and Nd^3^+ levels with the following rate equations.

#### Silicon nanoclusters

We consider both radiative *r *and non radiative *nr *transitions. The optical pumping power (in *m*^-3^) writes . The energy transfer between Si-ng and RE ions is described by a transfer coefficient *K *and equal to  at time *t*. This leads to the following rate equations:(4)(5)

### Rare earth ions Nd ^3+^

**Figure 2:**
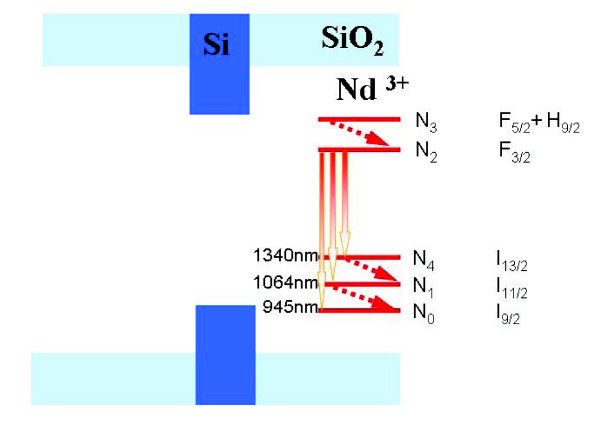
**Five level scheme of Nd^3+ ^ions**.

A five level scheme is adopted for the Nd^3+ ^ion in Figure 2[9,10].

We consider three radiative transitions (4*F*_3/2 _→ 4*I*_9/2_, *λ*_20 _= 945 nm; 4*F*_3/2 _→4*I*_11/2_, *λ*_21 _= 1064 nm; and 4*F*_3/2 _→ 4*I*_13/2_, *λ*_24 _= 1340 nm) and three non radiative transitions (4*F*_5/2 _- > 4*F*_3/2 _(*N*_3 _↦ *N*_2 _) 4*I*_11/2 _- > 4*I*_9/2 _(*N*_1 _↦ *N_0 _*) and 4*I*_13/2 _- > 4*I*_11/2 _(*N*_4 _↦ *N*_1_)).

The terms ,  and  correspond to the stimulated transitions 2 → 1, 2 → 0 and 2 → 4. The terms ,  and  correspond to the spontaneous transitions 2 → 1, 2 → 0 and 2 → 4.

The associated rate equations read:(6)(7)(8)(9)(10)

## Application to rib-loaded waveguide

In table 1, we collect the simulation parameters taken into account for the transitions. The lifetimes correspond to the experimental ones divided by the scaling factor 10^6^.

**Table 1:** Simulation parameters of the Si-ng and Nd^3+ ^transitions.

Transition	lifetime(*s*)	type	*ω_ij _*(*s*^-1^)	Δ*ω_ij _*(*s*^-1^)
Si →Si *	4 10^-11^	R	3.86 10^15^	4.4 10^14^

3 → 2	2.3 10^-16^	NR		

2 → 0	3 10^-10^	R	2.07 10^15^	1.38 10^14^

2 → 1	3 10^-10^	R	1.7 10^15^	1.39 10^14^

2 → 4	3 10^-10^	R	1.3 10^15^	1.33 10^14^

4 → 1	9.7 10^-16^	NR		

1 → 0	5.1 10^-16^	NR		

R and NR stand for radiative and non-radiative transitions respectively.

The transfer coefficient K estimated to ~ 10^-20 ^m^3 ^s^-1 ^[11] has also been scaled with the same factor 10^6^: *K *= 10^-14 ^m^3^.s^-1^. The amplitudes of the input pumping and signal electric fields have been taken equal to *E_pump _*= 10^7 ^V.m^-1 ^and *E_signal _*= 100 V.m^-1^.

After the time Fourier transform of both **E **and **H **fields, we deduce the *z *component of the pump () and signal () Poynting vectors (in W.m.^-2^)

**Figure 3:**
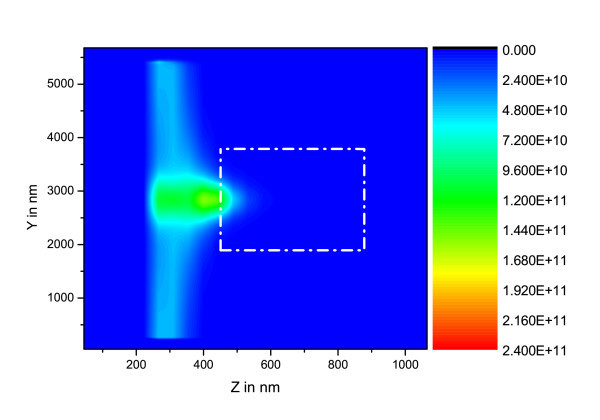
**(*yz*) maps of  in W.m^-2 ^for [Si-ng] = 10^25 ^m^-3^, the dashed-dot rectangle represents the waveguide rib**.

**Figure 4:**
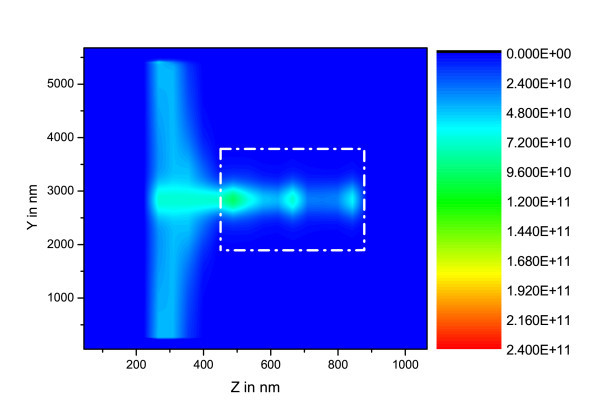
**(*yz*) maps of  in W.m^-2 ^for [Si-ng] = 10^24 ^m^-3^, the dashed-dot rectangle represents the waveguide rib**.

**Figure 5:**
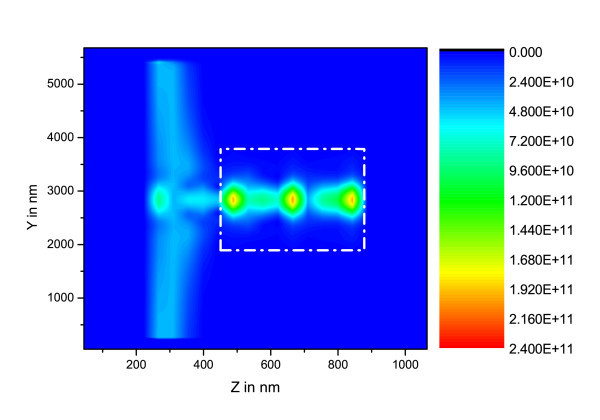
**(*yz*) maps of  in W.m^-2 ^for [Si-ng] = 10^23 ^m^-3^, the dashed-dot rectangle represents the waveguide rib**.

Three Si-ng concentrations have been investigated (N*_si _*= 10^25^, 10^24 ^and 10^23 ^m^-3 ^). In the initial states, only the ground level is populated. The corresponding (*xz*) maps () are plotted in Figures 3, 4 and 5.

In these figures, the different calculation box zones may be recognized: *i*) the Perfectly Matched Layer (PML) which lies in the area from the lefthand side between z = 0 nm and z = 180 nm, and from the righthand side between z = 890 nm and z = 1000 nm, *ii*) the plane containing the electromagnetic field source at z ~ 300 nm, *iii*) the FDTD zone which is located at about 180 nm from border of plot, the Gaussian beam impinging in the waveguide at z ~ 500 nm and the intensity propagating in waveguide from z ~ 500 nm to z ~ 900 nm.

On the basis of the parameters taken from experiments, these plots evidence the fact that for Si-ng concentrations above 10^24 ^m^-3^, the pumping wave does not reach the end of the waveguide. This concentration threshold corresponds to high experimental values [12], and is above the lower values leading to minimal optical losses [1].

**Figure 6:**
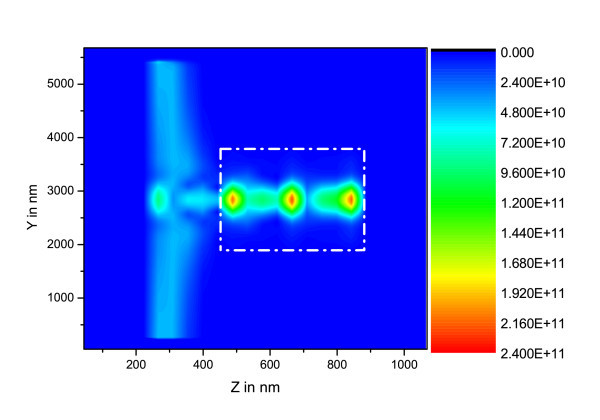
**(*yz*) map of  in W.m^-2 ^N*_Si _*5 10^22 ^m^-3^/ = 5 10^22 ^m ^-3^**.

In order to reduce the computing time, in addition to the scaling method, we start the calculations with Si-ng levels already populated at the maximum inversion rate, N*_Si _*=  = 5 10^22 ^m^-3^. Hence, for a given total Si-ng concentration of 10^23 ^m^-3^, this result (Figure 6) can be compared to the preceding one where N*_Si _*= 10^23 ^m^-3 ^and  (Figure 5). The propagation of the pump power within the waveguide seems to be similarly attenuated in both cases. The main difference occurs in the *N*_3 _level concentration which is directly populated from the excited  level. In case of maximum inversion rate, the stationary regime is reached and the concentration becomes equal to  10^18 ^m^-3^. In case of *N_Si _*= 10^23 ^m^-3 ^and  starting concentration, the *N*_3 _concentration does not reach a stationary regime and stays below several 10^17 ^m^-3^.

## Conclusion

We have investigated by means of ADE-FDTD method the electromagnetic field propagation in rib-loaded waveguides constituted of an active layer of Nd^3+ ^doped silicon rich silica stacked between pure silica bottom cladding and rib. This numerical method treats Nd^3+ ^and Si-ng levels rate equations (ADE) coupled to the Maxwell equations (FDTD). The extremely different specific times involved in the ADE (levels lifetimes ≈ 10 *μ*s) and in FDTD (electromagnetic wave periods ≈ 10^-15 ^s) have required the use of the scaling time method which allows reasonable computing time: the number of time iterations has been reduced by six orders of magnitude. In addition to this method, we have proposed to start the calculations with steady state Si-ng ground and excited populations. The numerical computation has been performed for several Si-ng concentrations. Therefore we can infer that the pumping wave propagation (*λ_pump _*= 488 nm ) is possible for [Si-ng] ≤ 10^24 ^m^-3 ^in agreement with experimental loss measurements. The upper Nd^3+ ^level reaches its stationary value predicted with the analytical solution of the steady state rate equations.

## Competing interests

The authors declare that they have no competing interests.

## Authors' contributions

CD and JC conceived the calculation code, AF carried out most of the calculations, OD performed the optical measurements on our samples and FG conceived the whole project. All authors read and approved the final manuscript.
